# The Evaluation of the Clinical Effects of Botulinum Toxin on Nocturnal Bruxism

**DOI:** 10.1155/2017/6264146

**Published:** 2017-07-05

**Authors:** Fatih Asutay, Yusuf Atalay, Hilal Asutay, Ahmet Hüseyin Acar

**Affiliations:** ^1^Faculty of Dentistry, Department of Oral and Maxillofacial Surgery, Afyon Kocatepe University, Afyonkarahisar, Turkey; ^2^Vefa Diş Polikliniği, Afyonkarahisar, Turkey; ^3^Uzman DentaClinic, Bursa, Turkey; ^4^Faculty of Dentistry, Department of Oral and Maxillofacial Surgery, Bezmialem Vakıf University, İstanbul, Turkey

## Abstract

**Objectives:**

Nocturnal bruxism can be managed by botulinum toxin (Botox®) in patients who have not responded to conservative treatment. The aim of this study was to evaluate the efficacy of botulinum toxin A (BTXA) in the treatment of nocturnal bruxism.

**Material and Methods:**

The retrospective study comprised 25 female patients, aged 23–55 years (mean 35.84 ± 8.41 years). All patients received a single injection of BTXA in the right and left masseters. Evaluation was made by Visual Analogue Scale (VAS) values, complaint duration, onset of effect, and duration of effectiveness.

**Results:**

BTXA produced significant improvements in pain scores. Only 2 adverse events (8%) were recorded.

**Conclusion:**

BTX-A is effective in the treatment of nocturnal bruxism.

## 1. Introduction

Nocturnal bruxism (NB) is defined as abnormal maxillomandibular activity during sleep, characterized by grinding and clenching of the teeth [1, 2]. NB can lead to wear on the teeth, dental prostheses/implant failure, tooth sensitivity, pain in the teeth, jaw, masticatory muscle, and temporomandibular joint (TMJ), neck pains and headache, periodontal disease, oral or facial pain, and potentially tooth loss [3, 4]. These problems can be associated with the unconscious and intense contractions of the temporal and masseter muscles during sleep [5]. There are various treatment techniques for the management of NB such as oral splint, behavioral approaches, and medications (muscle relaxants, botulinum toxin) but none is widely accepted [4].

Botulinum toxin (Botox) is an exotoxin produced by the bacterium* Clostridium botulinum*, which blocks acetylcholine release from cholinergic nerve endings into the neuromuscular junction, thereby causing inactivity of muscles or glands [6]. Botulinum neurotoxin types A (BTXA) and B (BTXB) are approved by the United States Food and Drug Administration (FDA) including cervical dystonia, severe primary axillary hyperhidrosis, strabismus, blepharospasm, hemifacial spasm, and glabellar wrinkles for BTXA and cervical dystonia for BTXB.

BTXA has been used in the treatment of cosmetic and noncosmetic conditions such as tremor, hemifacial spasm, temporomandibular joint dysfunction, bruxism, masticatory myalgias, sialorrhea, hyperhidrosis, and headache [7].

Botox injections are directly applied into the masseter and temporalis muscles to relax these muscles. The clinical effects are typically seen on the first to third days after the injection, followed by one to two weeks of maximum effect, and the typical duration of the effect is three to four months [8].

In two comprehensive review studies, Tinastepe et al. [1] and Manfredini et al. [2] suggested that there is not enough evidence to use botulinum injection in the treatment of bruxism. The purpose of the present study was to investigate the potential performance of BTXA on nocturnal bruxism and to share this clinical experience.

## 2. Materials and Methods

In the period 2014-2015, a retrospective data analysis was made of the data of 25 female patients who underwent onabotulinum toxin A (Botox, Allergan, Inc., Irvine, CA, USA) injections into the masseter muscle for clinically diagnosed nocturnal bruxism. The patients enrolled in the study had all failed to respond to conservative treatment. Exclusion criteria were temporomandibular disorders, pregnancy, active drug use, and psychological therapy.

For evaluation of postoperative pain, Visual Analogue Scale (VAS) forms were completed by the patients scoring the degree of pain between 0 (absence of pain) and 10 (maximum pain) before the injection and then at 2 weeks, 1 month, 3 months, 4 months, and 6 months after the injection.

The following data were recorded for all patients: maximum mouth opening changes, duration of the complaint before the injection, muscle weakness before injection, the time the first effects were seen, the time when the effectiveness started to be lost, and difficulty experienced in speaking, swallowing, or chewing.

This study was based on routine service documents of NB patients. However, permission to analyse the data was sought from the management of the Dentistry Hospital of Afyon Kocatepe University. All procedures followed were in accordance with the ethical standards of the committee responsible for human experimentation (institutional and national) and with the Helsinki Declaration of 1975, as revised in 2008. Written informed consent was obtained from all the study participants.

### 2.1. Procedure

For all patients, 100 mouse units (MU) of onabotulinum toxin A (Botox, Allergan, Inc., Irvine, CA) were diluted in 2 ml of saline.

In all patients, a dose of 20 MU of BTXA was injected into a single masseter muscle using a 0.5 mL insulin syringe at four points (5 MU/site). The patient was requested to clench the masseter muscle, and the injections were applied to the origin, insertion, anterior, and posterior parts of the muscle.

### 2.2. Statistical Analysis

Statistical analysis was performed with the Statistical Package for the Social Science Program (version 20.0, SPSS Inc., Chicago, USA). Differences in individual parameters were tested using the repeated measures test ANOVA and post hoc Friedman and Wilcoxon test for abnormally distributed variables (pain). A value of *p* < 0.05 was considered statistically significant.

## 3. Results

Evaluation was made of the data of 25 female patients with a mean age of 35.84 ± 8.41 years (range, 23–55 years). All the study variables are shown in Tables 1 and 2.

One patient had pain in the injection points. No adverse effects or dry mouth (injection into parotid gland) was reported in any other patients.

There were no significant changes in respect of the maximum mouth opening. Only 2 patients (8%) had no significant improvements in the pain scores after treatment.

The mean duration of the complaint before injection was 8.84 ± 4.57 years, the time that the effects were first seen was 12.24 ± 2.02 days, and the time that the loss of effectiveness started was 4.76 ± 1.01 months (Table 3).

The differences between two-week–four-month and one-month–three-month postoperative pain values were not statistically significant. However, differences between all other times were statistically significant (Table 4).

## 4. Discussion

In the present study, there were significant differences in reduction of pain and bruxism activity.

Van Zandijcke and Marchau [9] used botulinum toxin for the first time in the treatment of bruxism of a young woman with a brain injury, and a marked reduction was observed. Thereafter, botulinum toxin was used for the treatment of bruxism with different origins such as cranial-cervical dystonia [10], Huntington's disease [11], autism [12], and amphetamine addiction [13].

To date, only BTXA and BTXB serotypes have been approved by the US Food and Drug Administration (FDA) for clinical use including cervical dystonia, severe primary axillary hyperhidrosis, strabismus, blepharospasm, hemifacial spasm, and glabellar wrinkles for BTXA and cervical dystonia for BTXB.

Lang [14] reported that BTXA showed better and longer pain relief than BTXB. Moreover, BTXA had fewer side-effects than BTXB [14]. In our clinic, BTXA has been safely used for the treatment of bruxism, masseteric hypertrophy, and sialorrhea.

Although the pathogenesis of bruxism remains unclear, it is generally accepted that the etiology is multifactorial in nature. The occurrence of nocturnal bruxism suggests that it may occur as a result of possible physical or psychological conditions (emotional stress, anxiety, aggressive and hyperactive personality types, malocclusion, sleep problems such as sleep apnea, earache, headache, and tooth ache, the side-effects of some psychiatric medications, etc.) [13, 15].

The treatment of myofascial pain can be difficult for both clinicians and patients because most of these disorders involve multiple components such as somatic, neurogenic, and psychogenic components. Moreover, it can be resistant to conventional medical or behavioral therapy. There is no consensus on how the effects of Botox reduce pain. Some hypotheses have suggested changes in levels of muscle nociceptor sensitizers, nonacetylcholine neurotransmitters, and acetylcholine at autonomic synapses [16–18] and decreased motor neuron activity [19].

Nocturnal bruxism may lead to pain in the head, neck, jaw, teeth, and temporomandibular joint (TMJ). However, conservative treatments may be limited in the resolution of this problem. In the past few years, the use of Botox therapy has become a promising source for the management of myofascial pain [20]. The findings related to pain in the current study showed a significant difference before and after the injection, which is consistent with the findings of Sidebottom AJ et al. [21], Guarda-Nardini et al. [22], and Tan et al. [23].

In a study by Lee et al. [24], it was reported that BTXA injection reduced the number of bruxism events during sleep and it was therefore suggested that BTXA injection could be used as an effective treatment for nocturnal bruxism. Similarly, Santamato et al. [25] reported that neck pain related to nocturnal bruxism can be treated with BTXA. In the study by Santamato et al. [25], the dose of BTXA injected into each masseter muscle was 40 MU and 25 MU was injected into each temporalis muscle. Moreover, electromyography was used to measure to the changes of masseter and temporalis muscle hyperactivities and VAS was used to measure to pain [25]. In the present study, 20 MU BTXA was injected into each masseter muscle and positive results were reported.

Kesikburun et al. [26] reported a 21-year-old male patient with traumatic brain injury, who was successfully treated with BTXA for bruxism. According to our clinical experience, BTXA can be used safely for the treatment of bruxism.

The maximum mouth opening may be increased after injection of BTXA. Sidebottom et al. [21] and Guarda-Nardini et al. [22] reported that the interincisal distance can be induced with BTXA injection. However, the results of the current study do not match this conclusion. The differences may be due to most of the patients having no previous complaint of limited mouth opening or they may have been due to the small sample size.

The limitations of the present study include the small sample size and that there was no information about temporal injection or difference between genders.

In conclusion, Botox therapy seems promising and beneficial in the treatment of nocturnal bruxism, although several limiting factors such as high cost and the necessity for repeated injections prevent its widespread use. When there has been no response to conservative treatment methods, botulinum toxin may be an alternative and effective treatment for nocturnal bruxism and masticatory pain. Future studies with a larger number of patients are required to confirm the conclusions reached in the present study.

## Figures and Tables

**Table 1:** The demographic data, pain scores, the time of the onset of the effect, the time of the onset of the loss of effect, and the duration of the complaint of all the patients.

Patient	Age	VAS Scores						Onset of effect (day)	Onset of lose of effect (month)	Duration of complaint (year)
		Pre-op	2.w	1.m	3.m	4.m	6.m			
1	25	8	1	1	0	2	6	10	4	1
2	23	5	1	1	3	4	5	13	3	4
3	24	7	5	1	1	1	7	14	6	1
4	29	8	3	1	0	0	3	14	5	8
5	55	8	7	6	6	7	8	15	4	12
6	36	6	2	1	1	1	2	12	5,5	5
7	31	7	0	0	0	1	3	10	5	15
8	26	8	2	4	7	7	7	15	2,5	9
9	40	9	5	2	2	2	4	13	5	11
10	41	6	3	1	1	1	4	12	5	7
11	36	7	3	0	0	2	4	11	6	9
12	34	4	0	0	0	0	1	10	5	4
13	37	6	2	1	1	1	2	14	5	5
14	29	7	2	1	1	2	3	12	4,5	8
15	48	7	1	0	0	0	1	13	5,5	10
16	43	6	3	2	2	2	3	15	5	3
17	51	8	1	0	0	2	3	9	4	12
18	46	8	3	1	1	1	2	9	4	14
19	33	9	4	1	1	1	3	11	5	18
20	32	7	0	0	0	1	2	8	4	13
21	37	7	2	0	0	1	2	14	4	7
22	29	9	2	1	1	1	1	13	6	6
23	42	8	7	7	7	7	7	13	5	15
24	35	6	0	0	0	0	1	12	5,5	13
25	34	7	1	0	0	0	1	14	5	11

*Abbreviations*. VAS: Visual Analogue Scale, BTXA: Botulinum Toxin A, w: week, m: month.

**Table 2:** The characteristics of the patients according to the pain scores.

	VAS
Before injection	7.12 ± 1.236 (4–9)
2nd week	2.40 ± 1.979 (0–7)
1st month	1.28 ± 1.815 (0–7)
3rd month	1.40 ± 2.141 (0–7)
4th month	1.88 ± 2.128 (0–7)
6th month	3.40 ± 2.141 (1–8)

**Table 3:** The characteristics of the patients according to age, onset of effect, onset of loss of effect, and duration of complaint.

	Age	Onset of effect (day)	Onset of loss of effect (month)	Duration of complaint (year)
Mean ± SD	35.84 ± 8.41	12.24 ± 2.02	4.76 ± 1.01	8.84 ± 4.57
Min–max	23–55	8–15	2–6	1–18

**Table 4:** Test statistics^a^. ^a^Wilcoxon signed ranks test. ^b^Based on positive ranks. ^c^Based on negative ranks.

	vas2w-vaspi	Vas1m-vaspi	Vas3m-vaspi	Vas4m-vaspi	Vas6m-vaspi

*Z*	−4,390^b^	−4,410^b^	−4,395^b^	−4,392^b^	−4,124^b^
Asymp.Sig. (2-tailed)	,000	,000	,000	,000	,000

	vas1m-vas2w	vas3m-vas2w	Vas4m-vas2w	vas6m-vas2w	

*Z*	−3,257^b^	−2,751^b^	−1,629^b^	−2,644^c^	
Asymp.Sig. (2-tailed)	,001	,006	,103	,008	

	vas3m-vas1m	vas4m-vas1m	vas6m-vas1m	vas4m-vas3m	

*Z*	−,736^c^	−2,656^c^	−4,234^c^	−2,762^c^	
Asymp.Sig. (2-tailed)	,461	,008	,000	,006	

	vas6m-vas3m	Vas6m-vas4m			

*Z*	−4,148^c^	−4,239^c^			
Asymp.Sig. (2-tailed)	,000	,000			

vaspi: VAS score at preinjection; vas2w: VAS score at 2 weeks (after injection); vas1m: VAS score in first month; vas3m: VAS score in third month; vas4m: VAS score in fourth month; vas6m: VAS score in sixth month.
